# An approach to reduce inhaler errors using Donabedian's triad

**DOI:** 10.3389/fmedt.2024.1494089

**Published:** 2024-11-12

**Authors:** Dorothy May Isip Cruz, Manjush Karthika, Ashraf Alzaabi

**Affiliations:** ^1^ Faculty of Medical and Health Sciences, Liwa College, Abu Dhabi, United Arab Emirates; ^2^ Department of Internal Medicine, College of Medical and Health Sciences, UAE University, Al Ain, United Arab Emirates; ^3^ Division of Respirology, Zayed Military Hospital, Abu Dhabi, United Arab Emirates

**Keywords:** inhaler errors, Donabedian’s triad, structure, process, outcome

## Abstract

Inhaler errors inversely affect the outcome of respiratory diseases. Inhaler devices, such as the metered-dose inhalers (MDI) and dry powder inhalers (DPI), are commonly used in treating respiratory diseases like asthma and chronic obstructive pulmonary disease (COPD), and incorrect use of these devices can result in suboptimal treatment outcomes, increased probabilities of hospitalizations or admissions, and poorer quality of life. Patient related factors to inhaler errors include age, cognitive and physical abilities, education, language barriers, and preferences. Device-related factors such as inhaler design and operational complexity can also lead to errors. Finally, factors related to healthcare professionals (HCP) such as competency, level of knowledge in disease and inhaler device and availability to educate patients, can play a role in inhaler error. Quality management is a potential solution to this problem. Quality improvement strategies towards addressing inhaler misuse can increase patient satisfaction and improve patient outcomes. Donabedian's triad, which includes structure, process, and outcome can be utilized in developing a framework for reducing inhaler errors. Institutional solutions are more towards the structural and process changes in the triad, such as HCP training, checklists on training efficacy, provision of action plans, and availability of staff to educate and train patients. Patient-centered solutions focus more on process and outcome domains, such as improvement in lung functions, patient education, re-assessment and re-education of inhaler techniques, and adherence to treatment regimen. By focusing on structural and process domains, the quality of care can be enhanced, resulting in improved outcomes.

## Background

Therapeutic aerosols are medicated particulates that can be directly delivered to the airways and the lungs through the process of inhalation by devices like pressurized metered-dose inhalers (pMDIs), dry powder inhalers (DPIs) and nebulizers (1, 2). Their use has skyrocketed since the invention of the first pMDI by Riker Laboratories back in 1956, immediately gaining recognition worldwide, while other devices such as DPIs, soft mist inhalers (SMI) and nebulizers were evolving several years later (3).

In current times, there is an abundance in aerosol therapy devices, especially with inhalers, which vary in design based on the ease of delivering the drug, patient preference, or ability to use the device, correlating with the patient's capability to achieve a good inhaler technique (4). However, there has been a growing concern of inhaler misuse, with the bulk of them stemming from inadequate inhaler methods (5, 6). One of the studies being the CRITical Inhaler mistaKes and Asthma controL (CRITIKAL) study in 2017, which investigated inhaler techniques of 3,660 asthma patients, reporting about their exacerbations in relation to specific inhaler errors (7). The most critical error upon using a DPI was poor inspiratory effort in both Turbohaler and Diskus (OR 1.30 [1.08–1.57] and 1.56 [1.17–2.07] respectively, 95% CI) as DPIs require a fast inhalation due to their flow-dependent release of dose, as well as the failure to seal the lips around the mouthpiece, whereas the most critical error upon using an MDI was the actuation of the dose before inhaling (OR, 1.55; 95% CI, 1.11–2.16), all of which has resulted in poor control of asthma (7).

Another notable systematic review was done by the Aerosol Drug Management Improvement Team (ADMIT) in 2016, gathering 144 literatures from 1975 to 2014 on common MDI and DPI errors (8). Similarly to the CRITIKAL study, frequent MDI errors were minimal breath hold after inhalation (46%; 95% CI, 42%–49%), inspiratory rate and/or depth (44%; 40%–47%), and coordination (45%, 41%–49%), whereas frequent DPI errors were minimal breath hold after inhalation (37%, 33%–40%), inadequate or no exhalation before inhalation of drug (46%, 42%–50%), and poor preparation (29%, 26%–33%) (8). These are just some of the abundant literatures that emphasize patients’ inhaler misuse and a desperate call for a solution.

Despite the efforts made, such as the implementation of regular training programs for both patients and health-care providers, educational materials, digital resources, and device specifications like breath-actuated MDI, spacers, etc. for easier inhalation (6), there are still increasing occurrences of poor aerosol therapy use on a global scale, resulting in insufficient drug delivery to the airways, and eventually poor exacerbation control and decreased quality of life.

Quality management is an important element in healthcare provision as it reflects the efficacy of a systematic approach to certain medical events, patient outcomes in relation to care provided, continuous evaluation of quality provided, and many more. With the incorporation of a quality management program in relation to inhaler use, the probability of inhaler errors may be minimized, and eventually benefit the patient in the long run. This narrative article will further emphasize the numerous factors that affect the effectiveness of therapeutic inhaler use, as well as a proposed approach towards minimizing inhaler errors through the implementation of a quality management program.

## Factors that result in inhaler errors

The goal of aerosol therapy is to improve patient condition by minimizing or controlling symptom exacerbation, being individually modified for different patient requirements. And yet, there are evident studies suggesting the fact that inefficient inhaler instruction delivery with unsatisfactory demonstrations of techniques, stemming from both the healthcare professional and the patient, leads to increased likelihood of errors in operating inhalers, and eventually, poor exacerbation control (7, 8). Any factor that compromises the delivery of therapeutic aerosols resulting in insufficient drug deposition is considered as an inhaler-related error. This can be further classified into two categories known as critical and non-critical.

Critical errors are likely to compromise the delivery of adequate medication to the lungs whereas non-critical errors tend to limit the amount of drug that reaches the lungs compared to what can be achieved with good handling techniques (9, 10). Both critical and non-critical errors can occur due to various factors associated with either the patient, the device, or healthcare-professionals, all of which can result in, but are not limited to, the so-called potential inhaler errors, listed in Table 1.

**Table 1:** List of factors that affect effective inhaler usage. (Acknowledgement: References 7, 9, 11–14).

Patient-related factors
Age Gender Health beliefs Underlying disease status Income Language barrier Adherence to medication Physical capabilities Lack of education or understanding Personal preference
Device-related factors
Dispensing mechanism (e.g., propellant, compressed air, mechanical) Formulation of the medication (e.g., dry powder, solution) Single or multiple dose inhaler Dose preparation (e.g., DPIs) Common errors based on type of device used: • pMDI: Inadequate synchronization between actuation and inhalation Incomplete exhalation prior to inhaling Not holding their breath post-inhalation Rapid or shallow inhalation Triggering the device more than once within a single breath Poor mouth seal • DPI: Suboptimal dose loading Exhaling into the device Using the DPI when it is still damp after cleaning Device-specific issues such as failing to place capsule in Breezhaler, or failing to rotate lever in Diskus etc. • SMI: Incomplete exhalation away from the device Inability to hold their breath up to 10 s Poor hand-mouth coordination Failure to maintain device upright
Healthcare professional-related factors
Inappropriate device prescription Lack of information regarding different types of inhalers Poor or no technique education to the patient Prescription of multiple devices Limited time for patient education Lack of interest

### Patient-related factors

Effective inhaler use is influenced by multiple patient-related factors, with one of the main ones being their physical capabilities. Physical conditions, like arthritis for example, can hinder patients from effective inhaler manipulation, despite knowing the proper technique (15).

Another factor mentions the patient's health belief, impacting their adherence to regular inhaler use, where those who unequivocally believe that inhaler use is an integral part of their health are more likely to maintain adherence to their medication (9). In the medical field, adherence is explained as the extent to which a patient comprehends and accepts clinical instructions provided by healthcare practitioners, implying their willingness to follow prescribed treatment (16). On the opposite side, nonadherence implies refusal or non-compliance with treatment. Unintentional nonadherence occurs when regimens or information about device usage is misunderstood due to possible miscommunication, linguistic limitations, or forgetfulness, whereas intentional nonadherence is the more deliberate act of declining treatment due to personal beliefs of redundancy, inefficacy, or that the disease poses a threat to one's quality of life (17–19).

On the other hand, patients’ preferences also play a role in their adherence to an inhaler in terms of operation, such as straightforward instructions for use, activation, and cleaning, portability, forms, and longevity, as well as mouth sensations like aftertastes and discomfort (9, 20). In addition, the patient's capacity to inhale and hold a breath has an impact on how much benefit they receive from treatment. DPI medicines, for example, require the patient to produce a high peak inspiratory flow to deposit inside the lungs, and failure to do so would possibly result in an ineffective deposition that remains within the oropharynx (21). Furthermore, upon prescription of a new device, familiarity with other inhalers should be taken into consideration due to a possible learning effect presented by patients where they might have predetermined assumptions of similar applications towards subsequent devices through past encounters, however, that remains relevant if the device comprises of corresponding features, otherwise, it could be challenging to learn a completely different device with new functionalities (22). In the CRITIKAL study it was reflected that certain inhaler mistakes, such as inadequate coordination and failure to exhale before inhaling, were linked to poorer asthma outcomes, such as more frequent exacerbations. The study emphasized the significance of patient education in minimizing these errors, as better technique can result in improved disease management and reduced hospitalizations (7). This approach shifts the focus to the patient, aligning with the idea of a patient-driven process rather than solely clinician-driven.

### Device-related factors

Aerosol delivery devices are continuously emerging as technology advances, being more equipped for improved drug deposition to the lungs while considering patient preference and coordination ability (2). At the same time, the abundance of device availability in the market can confuse healthcare providers in the usage of these devices, leaving room for more errors (4). Inhaler devices have a vast array of features from how the drug is allotted by the device (active or passive), to the composition of the medication (solution or dry powder, the nature and size of the particulates), dose requirement, refillable or temporary, and how the dosage is arranged for DPIs (9, 11, 23) Majority of the time, difficulties in using inhalers can be observed in children aged 4–16 years old and the older population, hence, it is vital to consider the patients’ particular practical abilities, such as agility, hand strength, lung capacity, and mouth-hand coordination when prescribing inhalers (24).

### Health care professionals as a factor

HCP like respiratory therapists, pulmonary physicians, pharmacists, and nurses are the primary points of contact with the patients, when it comes to inhaler devices and therapy. Participation of these HCP in the administration of aerosol therapy devices is a predominant element in obtaining successful execution of inhaler technique and maintaining quality technique over time (9). Errors such as lack of information regarding inhalers, prescribing numerous devices, limited time, and failing to evaluate inhaling technique may be presented by HCP (25). A systematic analysis was published regarding studies on HCP’ inhaler technique competency while using inhaler devices, extracting data from 6,304 HCP demonstrating inhaler techniques in both the pMDI and DPI with results showing that errors were more frequently made with a DPI concerning deficient preparation (89%; 95% CI, 82–95), incomplete exhalation prior to inhalation (79%; 95% CI, 68–87) and no breath-hold being done (76%; 95% CI, 67–84) (25). The evident poor technique within HCP have been indicative of them lacking interest in understanding aerosol therapy devices as educating patients on their usage was deemed less relevant to their professional expertise and possessed a pre-assumption that patients will be able to utilize their inhalers appropriately (25).

It is not surprising that a small percentage of patients, on the other hand, receive inhaler training, and even fewer people have their technique assessed over time (26). Therefore, it has been proposed that pharmacists are in an advantageous state to instruct and review patients’ inhaler methods considering they are the endmost HCP that patients encounter before independent use of their inhalers through a 2.5 min educational intervention with simultaneous verbal guidance and physical demonstration about inhaler use along with routine reassessment of techniques, bringing forth improved outcomes in patient exacerbations and strategies (26, 27).

When it comes to dealing with inhaler errors, quality is of utmost importance, despite where the problem originated from. An example would be the quality of service through the delivery of instructions for aerosol device usage from the clinician, the level of understanding that the patient acquired from the clinician, or patient skill demonstration post-instruction. Management of quality, starting from the recruitment of highly skilled personnel to patient satisfaction or outcome, is essential to eliminate or reduce chances of inhaler errors.

## Role of quality management

Quality management has a wide interpretation, varying from multiple institutes. Some claim that it is the accomplishment of tasks by healthcare professionals that eventually provide patient satisfaction, but the most widely acknowledged claim is the incorporation of a more strategic approach towards patient needs (28, 29). There is a subjective view in the term “quality”, but the overall focus remains towards prevention of errors rather than flawless patient experiences (28). The National Academy of Medicine claims that one's act is of quality if deemed “safe, effective, patient centered, timely, efficient, and equitable” (28).

• “Safe” is ensuring no harm is done or intended when providing care,
• “Effective” is being selective with acts that are known to be scientifically beneficial for the patient,
• “Patient-centered” is being attentive to patient preferences, needs and considering their values in decision-making,
• “Timely” is minimizing interruptions or delays in providing care,
• “Efficient” is correct utilization of energy and materials with minimal to no wastage, and finally,
• “Equitable” being non-discriminative when providing quality care (30).

The implementation of quality management programs in healthcare institutes is of high importance as they are significantly associated with hospital-level quality indicators (31).

In past literature, although not directly mentioned, quality has always been the main area of concern when aiming for desired patient outcomes, especially when it comes to inhaler use in relation to the previously mentioned factors. But, to understand the issue at hand, we must first understand how quality is measured, otherwise known as quality indicators, associated with inhaler use.

Quality indicators (QI) tend to have no fixed terms but appear to revolve within quantitative measures that can be used to monitor quality and evaluate the quality of important governance, management, clinical, and support functions that affect patient outcomes, or help identify areas for improvement (32, 33). A good indicator should be important, relevant, valid, reliable, meaningful and understandable, cost-friendly, and easily collected (33). A study done by To, et al. researched and concluded on the effective performance indicators of different domains for quality of care in asthma, gathered from a total of 1,228 articles, where some of the highest ranked performance indicators included asthma education from certified asthma educators, pulmonary function monitoring, asthma control monitoring, as well as the overall use and prescription of controller medications (34). Klomp et al. have also compiled a list of QIs based on the 1999 and 2001 Canadian consensus guidelines for asthma (35). With the help of these indicators, quality improvement strategies can be better planned out, determining which amongst them are essential for improving overall patient care, provision of services, and organizational tasks, and applying necessary changes if deemed necessary.

Alongside the gathering and utilization of these indicators, the process of implementing quality management programs requires commitment and quality training from health-care professionals for favorable patient outcome in terms of health and satisfaction, along with unceasing improvements in quality management systems, including internal audits, customer feedback, and preventative or reparatory actions compliant with hospital protocols (28).

Managing quality can be attained through the application of one of the various models, which have been put forth to aid in continuous quality improvement, with some well-known examples being the four-step quality model, Six Sigma, Lean, Kano model, Deming cycle, and many more. However, the concept that stands the test of time, in which the cornerstone of quality assessment is built, remains to be the Donabedian model.

### Donabedian's model to reduce inhaler errors

Donabedian's model or triad is a concept put forward in 1966 by Avedis Donabedian, a physician and a researcher in the field of healthcare quality improvement. His triad is used to describe the three components of healthcare quality: structure, process, and outcomes (36).

(1) Structure: One of the three components in the triad refers to the needful resources and infrastructure to offering healthcare, inclusive of physical facilities, equipment, appropriate staffing levels, and training and education programs, highly stressing on the importance of having the necessary resources and support in place to provide high-quality healthcare.
(2) Process: The process component of Donabedian's triad refers to the real-time delivery of healthcare services, including the procedures, hospital protocols, and practices used for both diagnostic and therapeutic purposes. This component emphasizes the magnitude of delivering healthcare services in a consistent, standardized, and evidence-based manner.
(3) Outcome: The third and most important component of the triad refers to the impact of healthcare on patients in relation to their health status, quality of life, and overall satisfaction with care, emphasizing the importance of measuring and improving patient outcomes, as the consequent goal of healthcare is to improve patient health and well-being.

There are no current literatures on implementation of Donabedian's model to improving quality management of inhaler misuse, however, there are several studies that illustrates the effectiveness of the mentioned model in the healthcare system (37–39).

Binder, et al. (37) utilized the application of Donabedian's triad for guiding any modifications to care provided in emergency departments (ED) during the first wave of the COVID-19 pandemic. As compared to the regular methods of attending a patient through the ED, they were able to recognize necessary structural changes to accommodate more patients while reducing the risk of spreading the virus to other patients and staff, attaining as such through expanding ED spaces, negative pressure rooms, presence of waiting room nurses, outdoor screening tents and coronavirus hotlines for screening purposes, personal protective equipment (PPE), and more. The process domain mentions patient screening methods, early notification of patients who are under investigation for COVID, PPE distribution and guidelines for differing care, launching of telehealth, etc. All of which were corresponding to number of evaluated patients, COVID positive patients, daily sick calls, return visits, mortality rates, hospitalizations, complications, percentages of infected staff and unattended patients. Using Donabedian's model, development of key changes in either structural or process domains were rapid, in synchrony with the emerging literature on COVID-19.

Moore and colleagues (38) integrated Donabedian's model to assess quality of trauma care within 57 adult trauma centers in Canada between 2005 and 2010. A total of 63,971 patients were evaluated, while the performance of their trauma systems in relation to structure, process and outcome were assessed, as well as identifying any significant correlation between the domains. The structural indicators used referred to a criteria checklist based on the American College of Surgeons Committee on Trauma which included commitment, trauma program and procedural protocols, process indicators were based on the adherence towards 15 process QIs, and lastly, outcome indicators relied on in-hospital mortality rates, readmissions, length of stay in hospital, and complications. Mean results in structural performances were 47.4/100 (95% CI, 43.6–51.1), adherence to QIs were 61%, and in outcomes, 4.7% of total patients have deceased, 6.6% were readmitted, 13.9% presented with at least one complication, and mean length of stay was 8.8 days. Furthermore, the article states moderately positive association between structure and process indicators, process QIs have a moderately negative association with outcome indicators, and strong association between outcome QIs, suggestive of validity in implementation of Donabedian's model in a trauma care system.

Yet another article using Donabedian's triad in quality improvement processes of intensive care units stated its significance in reflecting the culture of a unit and the related outcome and highlighted it as a comprehensive method to assess healthcare quality (39).

**Figure 1:**
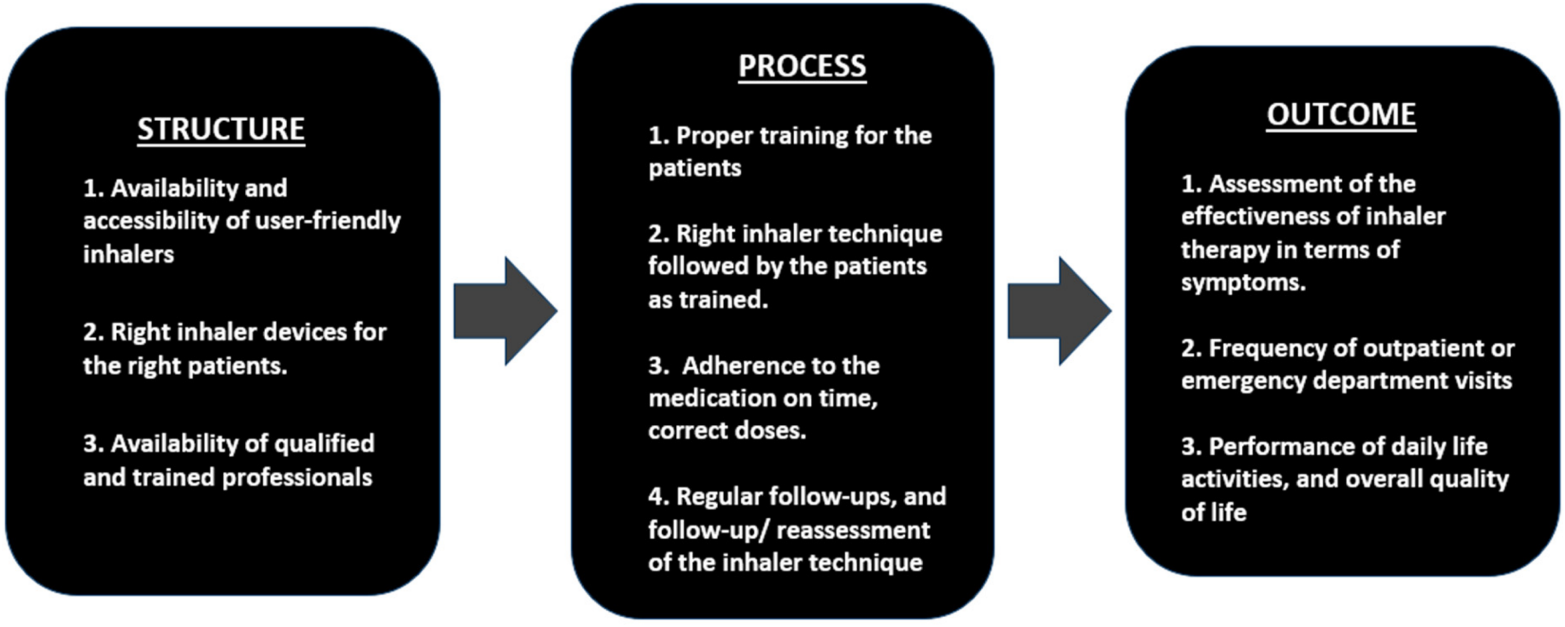
A schematic model of Donabedian's triad to reduce inhaler errors (40).

Although there is a paucity of studies specifically on the application of Donabedian's model in inhaler prescriptions, available literature provides a positive implication that considering a model based on the triad could further minimize occurrences of inhaler errors (40) (Figure 1).

Key aspects of structure in this model includes the availability of user-friendly and right inhaler devices, qualified staff, proper training for both healthcare professionals and patients on inhaler use and ensuring accessibility for patients with limited mobility or special needs.

The second component, process involves proper training of patients on correct inhaler technique, ensuring the adherence to inhalers on time with correct doses, and attend regular follow-up appointments and reassessment of their inhaler techniques.

The third and important component of the model, outcome evaluates the effectiveness of inhaler technique by evaluating the symptoms, frequency of outpatient or emergency visits, daily life activities, and overall quality of life. Validated methods like such as Asthma Control Tests, Chronic Obstructive Pulmonary Disease Assessment Test, St. George's Respiratory Questionnaire and Modified Medical Research Council Dyspnea scale are useful to evaluate the outcome.

## Approaches to minimize inhaler errors

### The structure

The initial section of Donabedian's triad, structure, emphasizes the foundation of quality healthcare, where, in context of preventing inhaler errors, the implementation of continuous quality evaluation at departmental levels reflects high quality healthcare. This remains a necessity for maintaining inhaler usage competency within medical practitioners as they bear a significant amount of responsibility when it comes to patients’ understanding of proper inhaler use (27). Basheti, et al. have conducted a study in Jordan, assessing random specialists on inhaler techniques before and after inhaler training workshops on Diskus, Turbuhaler and MDI, concluding that optimal inhaler technique can be obtained within 2-hour hands-on workshops and maintained for a long period of time [mean scores between attendees and non-attendees of 7.64 vs. 5.99 (*P* < .001)] (41). This shows that it is critical that HCP are proficient enough to have a broad range of knowledge about both the operational and conceptual working of a device be trained and assessed by professionals for correct inhaling methods, to which they could successfully demonstrate towards educating patients. Alongside this, certain checklists, process descriptions or a systematic list of physical steps that demonstrate attempts made by HCP to train the patients can be incorporated to gather information and evaluate the efficiency of their patient education (22). HCP need to be dedicated with educating and assisting their patients on inhaler usage as patients tend be imperceptive on dealing with their respiratory symptoms, which can be highly attributed with a patient's belief that using an inhaler is easy (42–45). Therefore, to minimize the frequency and severity of exacerbations, a regular, comprehensive disease education to patients with individually adjusted linguistics should be incorporated in their daily regimen (46, 47).

HCP like pharmacists play a crucial role in providing education and ensure proper inhaler technique, leading to better disease management. Studies have shown that pharmacist-led interventions significantly enhance inhaler use and disease control. For instance, Armour et al. showed reduced hospital admissions and improved quality of life with pharmacy-based asthma care programs (48), while Petite et al. summarized that pharmacists play a crucial role in educating and monitoring COPD patients on proper inhaler use (49). A personalized approach to COPD management involving pharmacists can enhance patient care, improve adherence, and reduce inhaler misuse in a cost-effective manner.

Apart from knowing how to educate the patient, HCP must also be able to prescribe inhalers suitable for patient requirements or capabilities, as mentioned in patient-related factors. The more satisfied a patient is with their device, taking into consideration their preferences, the higher the likelihood of adherence to treatment, resulting in better symptom control and reduced hospital visits, as well as patient questionnaires to examine their satisfaction upon the prescribed inhaler and if there are any clinical improvements (11, 50). Adjuncts, such as a spacer, must be provided to patients with poor hand-to-mouth coordination, especially in children (11).

Spacers, or valved holding chambers, help to improve airway deposition of medication delivered through a pMDI, following actuation with a slow, deep inspiration. The use of a spacer overcomes the difficulty of hand-to-mouth coordination, allowing more time for the patient to comfortably take in their medication with tidal breaths (51). Studies have shown that children and/or adults with asthma had better bronchodilatation effects (7, 52), better lung deposition of the drug (53), reduced events of wheezing with decreased need of hospitalization (54, 55), and better device technique (56).

To add on, several obstructive disease action plans have been constructed to evaluate patients’ management plans on a normal basis or at events of deterioration, also known as the Asthma or COPD Action Plans. These are published by institutes like the National Heart, Lung and Blood Institute, Lung Foundation Australia, and the Canadian Thoracic Society in an attempt to minimize exacerbations, comprising of color-coded zones with instructions or guidelines to follow as advised by the attending physician. These zones are categorized into three; the green zone for normal patient conditions, yellow zone for worsening symptoms of dyspnea, cough, chest tightness, changes in peak flow measurements, etc., and the red zone for immediate requirement of medical attention (57–59). The incorporation of action plans in asthma and COPD have been shown to be beneficial in both adult and pediatric sections with fewer exacerbations, emergency visits to hospitals, and better quality of daily living (60–63).

### Process and outcome- A patient-centered approach

The process of inhaler technique assessment, patient education, treatment adherence, routine follow up and reassessments are another integral part of maintaining effective inhaler use.

As mentioned previously, the provision of action plans must be followed by the patient and their treatment is adjusted as necessary. This requires them to return to their physician as needed, depending on the severity of their case, with reassessment of their pulmonary functions. Process and outcome go together in these cases. Patients maintaining an optimal inhaler technique over time are of minimal guarantee, and the only way to ensure such is through a follow-up of their techniques by an HCP (22). The significance of technique re-evaluation needs to be emphasized more, as previous evidence reports only about 50% of HCP inquire about patient's inhaler technique (22, 64).

Simultaneously, regular, comprehensive disease education and delivery methods of device utilization need to be adjusted from patient to patient with different literacy capabilities, which is achievable through provision of a variety of educational formats. A 30 min session between the patient and the physician performing a teach-back method on inhaler technique has been proven to improve clinical outcomes in relation to their inhaler technique, despite varying levels of literacy (47), expressing how patient feedback is highly beneficial. This can be attained through means of a sequential video guide, feedback monitors or add-ons, or through simple, yet constructive physical and verbal feedback (22).

In addition to the advancement of technology comes the development of digital monitoring devices known as “smart inhalers” coming in a variety of forms, including instant feedback within the device itself, a mountable item that detects specific operations such as priming and actuation, and applications on smartphones that are linked to sensors on the inhaler (37). These facilitate recordings of patient information such as the number of doses taken or remaining for the day, alerts about when to take a dosage, and real-time assessment of inspiratory effort and technique (37, 38). Moreover, environmental quality and allergen presence may be analyzed, allowing for the management of further symptom aggravation, and avoidance of triggers (38). Since the outcome depends on the impact of the structure and process, it focuses more broadly on evaluating the patient's experiences and feedback, reflecting the effectiveness of the model. In summary, Donabedian's triad, which includes structure, process, and outcome indicators, is proposed to have potential in reducing inhaler errors. By maintaining suitable inhaler devices, providing thorough training for healthcare providers and patients, and consistently monitoring and evaluating interventions, healthcare systems can nurture a multifaceted approach to promote systematic and sustainable pathways to improve patient outcomes.

## Challenges in the out-of-hospital settings

Ensuring consistent care for inhaler use outside clinical settings is challenging, but Donabedian's model can potentially address these issues in the outpatient settings. By providing robust support systems and resources, such as clear educational materials and instructional videos, patients’ understanding of proper inhaler techniques can be significantly improved (65). Standardized training protocols during patient visits ensure consistent education on inhaler use by all healthcare providers, with follow-up calls or messages reinforcing this education. Personalized inhaler technique education by pharmacists has been shown to lead to sustained improvements in technique and asthma outcomes (66). Monitoring patient outcomes through regular assessments using patient-reported outcomes and remote monitoring tools is crucial to ensure effective self-management at home. Telemedicine can help overcome challenges in providing follow-up care by offering remote consultations for visual assessment of inhaler techniques and immediate feedback (67). Digital platforms with educational resources, reminders, and symptom tracking enhance patient engagement and self-management. Telemedicine also addresses geographical barriers, enabling consistent care in remote or underserved areas, as seen during the COVID-19 pandemic (68).

In a wider context, training local community health workers with inhaler techniques and technology, to an extent may help to improve healthcare services in remote areas. Collaborative care models that integrate services across different levels of care can provide consistent support for patients. Encouraging healthcare systems to invest in telehealth infrastructure and community-based interventions through policy and advocacy can enhance access. By incorporating multidisciplinary teams and telemedicine into Donabedian's model, we can ensure patients receive necessary support for effective inhaler therapy management at home, leading to improved health outcomes.

## Conclusion

Prescribing inhalers to patients aims to reduce exacerbations and improve disease control. However, errors can occur due to factors related to patient, device, or healthcare professional. Quality management is crucial in minimizing inhaler errors, as evidenced by the literature on identifying errors and strategies to reduce negative outcomes. Continuous quality assessments, including evaluating skill proficiency and regular check-ups, should be implemented to support healthcare providers like respiratory therapists and pulmonary physicians in providing effective patient education. Donabedian's triad can guide the development of a quality assurance framework to analyze structural and process components of inhaler use, ultimately reducing hospital readmissions, promoting medication adherence and technique maintenance, and enhancing overall patient quality of life.

## Data availability statement

The original contributions presented in the study are included in the article/Supplementary Material, further inquiries can be directed to the corresponding author.

## References

[1] Sims MW. Aerosol therapy for obstructive lung diseases: device selection and practice management issues. Chest. (2011) 140(3):781–8. 10.1378/chest.10-2068 21896522 PMC3204795

[2] Lavorini F Fontana GA Usmani OS. New inhaler devices—the good, the bad and the ugly. Respiration. (2014) 88(1):3–15. 10.1159/000363390 24902629

[3] Stein SW Thiel CG. The history of therapeutic aerosols: a chronological review. J Aerosol Med Pulm Drug Deliv. (2017) 30(1):20–41. 10.1089/jamp.2016.1297 27748638 PMC5278812

[4] Haughney J Price D Barnes NC Virchow JC Roche N Chrystyn H. Choosing inhaler devices for people with asthma: current knowledge and outstanding research needs. Respir Med. (2010) 104(9):1237–45. 10.1016/j.rmed.2010.04.012 20472415

[5] Saunders KB. Misuse of inhaled bronchodilator agents. Br Med J. (1965) 1(5441):1037–8. 10.1136/bmj.1.5441.1037 14262196 PMC2166937

[6] Levy ML Hardwell A McKnight E Holmes J. Asthma patients’ inability to use a pressurised metered-dose inhaler (pMDI) correctly correlates with poor asthma control as defined by the global initiative for asthma (GINA) strategy: a retrospective analysis. Prim Care Respir J. (2013) 22(4):406–11. 10.4104/pcrj.2013.00084 24042172 PMC6442852

[7] Price DB Román-Rodríguez M McQueen RB Bosnic-Anticevich S Carter V Gruffydd-Jones K Inhaler errors in the CRITIKAL study: type, frequency, and association with asthma outcomes. J Allergy Clin Immunol Pract. (2017) 5(4):1071–81.e9. 10.1016/j.jaip.2017.01.004 28286157

[8] Sanchis J Gich I. Pedersen S, Aerosol Drug Management Improvement Team (ADMIT). systematic review of errors in inhaler use: has patient technique improved over time? Chest. (2016) 150(2):394–406. 10.1016/j.chest.2016.03.041 27060726

[9] Inhaler Error Steering Committee, Price D Bosnic-Anticevich S Briggs A Chrystyn H Rand C Inhaler competence in asthma: common errors, barriers to use and recommended solutions. Respir Med. (2013) 107(1):37–46. 10.1016/j.rmed.2012.09.017 23098685

[10] Usmani OS Lavorini F Marshall J Dunlop WCN Heron L Farrington E Critical inhaler errors in asthma and COPD: a systematic review of impact on health outcomes. Respir Res. (2018) 19(1):10. 10.1186/s12931-017-0710-y 29338792 PMC5771074

[11] Usmani OS. Choosing the right inhaler for your asthma or COPD patient. Ther Clin Risk Manag. (2019) 15:461–72. 10.2147/TCRM.S160365 30936708 PMC6422419

[12] Molimard M Raherison C Lignot S Balestra A Lamarque S Chartier A Chronic obstructive pulmonary disease exacerbation and inhaler device handling: real-life assessment of 2935 patients. Eur Respir J. (2017) 49(2):1601794. 10.1183/13993003.01794-2016 28182569

[13] Sanchis J Corrigan C Levy ML Viejo JL, ADMIT Group. Inhaler devices—from theory to practice. Respir Med. (2013) 107(4):495–502. 10.1016/j.rmed.2012.12.007 23290591

[14] Navaie M Dembek C Cho-Reyes S Yeh K Celli BR. Device use errors with soft mist inhalers: a global systematic literature review and meta-analysis. Chron Respir Dis. (2020) 17:1479973119901234. 10.1177/1479973119901234 31984767 PMC6985977

[15] Shirmanesh Y K Jones MD. Physical ability of people with rheumatoid arthritis and age-sex matched controls to use four commonly prescribed inhaler devices. Respir Med. (2018) 135:12–4. 10.1016/j.rmed.2017.12.014 29414447

[16] Rau JL. Determinants of patient adherence to an aerosol regimen. Respir Care. (2005) 50(10):1346–56; discussion 1357–9. 16185370

[17] Ari A. Patient education and adherence to aerosol therapy. Respir Care. (2015) 60(6):941–55; discussion 955–7. 10.4187/respcare.03854 26070585

[18] Haughney J Price D Kaplan A Chrystyn H Horne R May N Achieving asthma control in practice: understanding the reasons for poor control. Respir Med. (2008) 102(12):1681–93. 10.1016/j.rmed.2008.08.003 18815019

[19] Cochrane GM Horne R Chanez P. Compliance in asthma. Respir Med. (1999) 93(11):763–9. 10.1016/s0954-6111(99)90260-3 10603624

[20] Dekhuijzen PN Lavorini F Usmani OS. Patients’ perspectives and preferences in the choice of inhalers: the case for Respimat(®) or HandiHaler(®). Patient Prefer Adherence. (2016) 10:1561–72. 10.2147/PPA.S82857 27574405 PMC4993394

[21] Crompton GK Barnes PJ Broeders M Corrigan C Corbetta L Dekhuijzen R The need to improve inhalation technique in Europe: a report from the aerosol drug management improvement team. Respir Med. (2006) 100(9):1479–94. 10.1016/j.rmed.2006.01.008 16495040

[22] Bosnic-Anticevich SZ Cvetkovski B Azzi EA Srour P Tan R Kritikos V. Identifying critical errors: addressing inhaler technique in the context of asthma management. Pulm Ther. (2018) 4(1):1–12. 10.1007/s41030-018-0051-0 32026244 PMC6966926

[23] Salvi S Shevade M Aggarwal A Apte K Barne M Mohan MBV. A practical guide on the use of inhaler devices for asthma and COPD. J Assoc Physicians India. (2021) 69:8–26.

[24] Lavorini F Magnan A Dubus JC Voshaar T Corbetta L Broeders M Effect of incorrect use of dry powder inhalers on management of patients with asthma and COPD. Respir Med. (2008) 102(4):593–604. 10.1016/j.rmed.2007.11.003 18083019

[25] Plaza V Giner J Rodrigo GJ Dolovich MB Sanchis J. Errors in the use of inhalers by health care professionals: a systematic review. J Allergy Clin Immunol Pract. (2018) 6(3):987–95. 10.1016/j.jaip.2017.12.032 29355645

[26] Basheti IA Reddel HK Armour CL Bosnic-Anticevich SZ. Counseling about turbuhaler technique: needs assessment and effective strategies for community pharmacists. Respir Care. (2005) 50(5):617–23. 15871755

[27] Basheti IA Reddel HK Armour CL Bosnic-Anticevich SZ. Improved asthma outcomes with a simple inhaler technique intervention by community pharmacists. J Allergy Clin Immunol. (2007) 119(6):1537–8. 10.1016/j.jaci.2007.02.037 17433831

[28] Karthika M Sureshkumar VK Bennett A Noorshe AH Mallat J Praveen BM. Quality management in respiratory care. Respir Care. (2021) 66(9):1485–94. 10.4187/respcare.08820 34408082

[29] Aggarwal A Aeran H Rathee M. Quality management in healthcare: the pivotal desideratum. J Oral Biol Craniofac Res. (2019) 9(2):180–2. 10.1016/j.jobcr.2018.06.006 31211031 PMC6561897

[30] Institute of Medicine (US) Committee on Quality of Health Care in America. Crossing the Quality Chasm: A New Health System for the 21st Century. Washington (DC): National Academies Press (US) (2001). 10.17226/10027 25057539

[31] Weiner BJ Alexander JA Shortell SM Baker LC Becker M Geppert JJ. Quality improvement implementation and hospital performance on quality indicators. Health Serv Res. (2006) 41(2):307–34. 10.1111/j.1475-6773.2005.00483.x 16584451 PMC1702526

[32] Becker M Breuing J Nothacker M Deckert S Steudtner M Schmitt J Guideline-based quality indicators-a systematic comparison of German and international clinical practice guidelines: protocol for a systematic review. Syst Rev. (2018) 7(1):5. 10.1186/s13643-017-0669-2 29329578 PMC5767020

[33] Choong MK Tsafnat G Hibbert P Runciman WB Coiera E. Comparing clinical quality indicators for asthma management in children with outcome measures used in randomised controlled trials: a protocol. BMJ Open. (2015) 5(9):e008819. 10.1136/bmjopen-2015-008819 26351189 PMC4563246

[34] To T Guttmann A Lougheed MD Gershon AS Dell SD Stanbrook MB Evidence-based performance indicators of primary care for asthma: a modified RAND appropriateness method. Int J Qual Health Care. (2010) 22(6):476–85. 10.1093/intqhc/mzq061 20978002

[35] Klomp H Lawson JA Cockcroft DW Chan BT Cascagnette P Gander L Examining asthma quality of care using a population-based approach. CMAJ. (2008) 178(8):1013–21. 10.1503/cmaj.070426 18390944 PMC2276554

[36] Donabedian A. Evaluating the quality of medical care. Milbank Q. (2005) 83(4):691–729. 10.1111/j.1468-0009.2005.00397.x 16279964 PMC2690293

[37] Binder C Torres RE Elwell D. Use of the donabedian model as a framework for COVID-19 response at a hospital in suburban westchester county, New York: a facility-level case report. J Emerg Nurs. (2021) 47(2):239–55. 10.1016/j.jen.2020.10.008 33317860 PMC7831996

[38] Moore L Lavoie A Bourgeois G Lapointe J. Donabedian's structure-process-outcome quality of care model: validation in an integrated trauma system. J Trauma Acute Care Surg. (2015) 78(6):1168–75. 10.1097/TA.0000000000000663 26151519

[39] Savjani K Haseeb F Reay M. Measuring quality and outcomes in intensive care. Surgery. (2018) 36(4):196–200. 10.1016/j.mpsur.2018.01.008

[40] Karthika M Vanajakshy Kumaran S Beekanahaali Mokshanatha P. Quality indicators in respiratory therapy. World J Crit Care Med. (2024) 13(2):91794. 10.5492/wjccm.v13.i2.91794 38855272 PMC11155503

[41] Basheti IA Qunaibi EA Hamadi SA Reddel HK. Inhaler technique training and health-care professionals: effective long-term solution for a current problem. Respir Care. (2014) 59(11):1716–25. 10.4187/respcare.02671 24962222

[42] Azzi E Srour P Armour C Rand C Bosnic-Anticevich S. Practice makes perfect: self-reported adherence a positive marker of inhaler technique maintenance. NPJ Prim Care Respir Med. (2017) 27(1):29. 10.1038/s41533-017-0031-0 28439076 PMC5435088

[43] Price D David-Wang A Cho SH Ho JC Jeong JW Liam CK Time for a new language for asthma control: results from REALISE Asia. J Asthma Allergy. (2015) 8:93–103. 10.2147/JAA.S82633 26445555 PMC4590568

[44] Jahedi L Downie SR Saini B Chan HK Bosnic-Anticevich S. Inhaler technique in asthma: how does it relate to Patients’ preferences and attitudes toward their inhalers? J Aerosol Med Pulm Drug Deliv. (2017) 30(1):42–52. 10.1089/jamp.2016.1287 27676193 PMC5278803

[45] Micallef L. A review of the metered dose inhaler technique in asthmatic and COPD patients. Malta Med J. (2015) 27(1):22–8.

[46] Brown R. Asthma patient education: partnership in care. Int Forum Allergy Rhinol. (2015) 5(1):S68–70. 10.1002/alr.21596 26335838

[47] Kiser K Jonas D Warner Z Scanlon K Shilliday BB DeWalt DA. A randomized controlled trial of a literacy-sensitive self-management intervention for chronic obstructive pulmonary disease patients. J Gen Intern Med. (2012) 27(2):190–5. 10.1007/s11606-011-1867-6 21935752 PMC3270237

[48] Armour C Bosnic-Anticevich S Brillant M Burton D Emmerton L Krass I Pharmacy asthma care program (PACP) improves outcomes for patients in the community. Thorax. (2007) 62(6):496–502. 10.1136/thx.2006.064709 17251316 PMC2117224

[49] Petite SE Hess MW Wachtel H. The role of the pharmacist in inhaler selection and education in chronic obstructive pulmonary disease. J Pharm Technol. (2021) 37(2):95–106. 10.1177/8755122520937649 34752567 PMC7953076

[50] Levy ML Dekhuijzen PN Barnes PJ Broeders M Corrigan CJ Chawes BL Inhaler technique: facts and fantasies. A view from the aerosol drug management improvement team (ADMIT). NPJ Prim Care Respir Med. (2016) 26:16017. 10.1038/npjpcrm.2016.17; Erratum in: *NPJ Prim Care Respir Med*. (2016) **26**:16028. doi: 10.1038/npjpcrm.2016.28. 27098045 PMC4839029

[51] Vincken W Levy ML Scullion J Usmani OS Dekhuijzen PNR Corrigan CJ. Spacer devices for inhaled therapy: why use them, and how? ERJ Open Res. (2018) 4(2):00065–2018. 10.1183/23120541.00065-2018 29928649 PMC6004521

[52] Rachelefsky GS Rohr AS Wo J Gracey V Spector SL Siegel SC Use of a tube spacer to improve the efficacy of a metered-dose inhaler in asthmatic children. Am J Dis Child. (1986) 140(11):1191–3. 10.1001/archpedi.1986.02140250117046 3532764

[53] Newman SP Woodman G Clarke SW Sackner MA. Effect of InspirEase on the deposition of metered-dose aerosols in the human respiratory tract. Chest. (1986) 89(4):551–6. 10.1378/chest.89.4.551 3956281

[54] Castro-Rodriguez JA Rodrigo GJ. beta-agonists through metered-dose inhaler with valved holding chamber versus nebulizer for acute exacerbation of wheezing or asthma in children under 5 years of age: a systematic review with meta-analysis. J Pediatr. (2004) 145(2):172–7. 10.1016/j.jpeds.2004.04.007 15289762

[55] Rajkumar V Rajendra B How CH Ang SB. Wheeze in childhood: is the spacer good enough? Singapore Med J. (2014) 55(11):558–62; discussion 563. 10.11622/smedj.2014150 25631964 PMC4294002

[56] Crane MA Jenkins CR Goeman DP Douglass JA. Inhaler device technique can be improved in older adults through tailored education: findings from a randomised controlled trial. NPJ Prim Care Respir Med. (2014) 24:14034. 10.1038/npjpcrm.2014.34 25188403 PMC4373405

[57] Lung Foundation Australia. COPD Action Plan. Lung Foundation Australia (2023). Available online at: https://lungfoundation.com.au/resources/copd-action-plan/ (accessed January 20, 2024).

[58] Asthma Action Plan | NHLBI, NIH. www.nhlbi.nih.gov. (2020). Available online at: https://www.nhlbi.nih.gov/resources/asthma-action-plan-2020 (accessed January 25, 2024).

[59] Family Physician Airways Group of Canada. COPD Action Plan (2013). Available online at: https://www.fpagc.com/s/CTS_COPD_updated_Action_Plan_editable_PDF_2013.pdf (accessed January 20, 2024).

[60] Pegoraro F Masini M Giovannini M Barni S Mori F du Toit G Asthma action plans: an international review focused on the pediatric population. Front Pediatr. (2022) 10:874935. 10.3389/fped.2022.874935 35592848 PMC9113391

[61] Gibson PG Powell H Coughlan J Wilson AJ Abramson M Haywood P Self-management education and regular practitioner review for adults with asthma. Cochrane Database Syst Rev. (2003) (1):CD001117. 10.1002/14651858.CD001117 12535399

[62] McDonald VM Gibson PG. Asthma self-management education. Chron Respir Dis. (2006) 3(1):29–37. 10.1191/1479972306cd090ra 16509175

[63] Zemek RL Bhogal SK Ducharme FM. Systematic review of randomized controlled trials examining written action plans in children: what is the plan? Arch Pediatr Adolesc Med. (2008) 162(2):157–63. 10.1001/archpediatrics.2007.34 18250241

[64] Price D David-Wang A Cho SH Ho JC Jeong JW Liam CK Asthma in Asia: physician perspectives on control, inhaler use and patient communications. J Asthma. (2016) 53(7):761–9. 10.3109/02770903.2016.1141951 27096388

[65] Giraud V Roche N. Misuse of corticosteroid metered-dose inhaler is associated with decreased asthma stability. Eur Respir J. (2002) 19(2):246–51. 10.1183/09031936.02.00218402 11866004

[66] Basheti IA Armour CL Bosnic-Anticevich SZ Reddel HK. Evaluation of a novel educational strategy, including inhaler-based reminder labels, to improve asthma inhaler technique. Patient Educ Couns. (2008) 72(1):26–33. 10.1016/j.pec.2008.01.014 18314294

[67] Pinnock H Slack R Pagliari C Price D Sheikh A. Professional and patient attitudes to using mobile phone technology to monitor asthma: questionnaire survey. Prim Care Respir J. (2006) 15(4):237–45. 10.1016/j.pcrj.2006.03.001 16843066 PMC6730811

[68] Hollander JE Carr BG. Virtually perfect? Telemedicine for COVID-19. N Engl J Med. (2020) 382(18):1679–81. 10.1056/NEJMp2003539 32160451

